# Comparison of MAF-32 and a One-Pot Synthesized Superparamagnetic Iron Oxide/MAF-32 Composite for the Adsorption of Diclofenac

**DOI:** 10.3390/ma17102269

**Published:** 2024-05-11

**Authors:** Erick Ramírez, Daniela Carmona-Pérez, J. F. Marco, Karla R. Sanchez-Lievanos, Sergio A. Sabinas-Hernández, Kathryn E. Knowles, María P. Elizalde-González

**Affiliations:** 1 Centro de Química, Instituto de Ciencias, Benemérita Universidad Autónoma de Puebla, Ciudad Universitaria, Edif. IC7, Puebla Pue 72570, Mexico; daniela.carmo@alumno.buap.mx (D.C.-P.); maria.elizalde@correo.buap.mx (M.P.E.-G.); 2 Instituto de Química-Física Blas Cabrera, CSIC, c/Serrano, 119, 28006 Madrid, Spain; jfmarco@iqfr.csic.es; 3 Department of Chemistry, University of Rochester, Rochester, NY 14627, USA; ksanche6@ur.rochester.edu (K.R.S.-L.); knowles@chem.rochester.edu (K.E.K.); 4 Instituto de Física, Benemérita Universidad Autónoma de Puebla, Ciudad Universitaria, Apartado Postal J-48, Puebla Pue 72570, Mexico; ssabinas@ifuap.buap.mx

**Keywords:** pharmaceuticals, magnetic material, coordination polymer, water pollution, Metal Azolate Framework

## Abstract

The global presence of pharmaceutical pollutants in water sources represents a burgeoning public health concern. Recent studies underscore the urgency of addressing this class of emerging contaminants. In this context, our work focuses on synthesizing a composite material, Fe_x_O_y_/MAF-32, through a streamlined one-pot reaction process, as an adsorbent for diclofenac, an emerging environmental contaminant frequently found in freshwater environments and linked to potential toxicity towards several organisms such as fish and mussels. A thorough characterization was performed to elucidate the structural composition of the composite. The material presents magnetic properties attributed to its superparamagnetic behavior, which facilitates the recovery efficiency of the composite post-diclofenac adsorption. Our study further involves a comparative analysis between the Fe_x_O_y_/MAF-32 and a non-magnetic counterpart, comprised solely of 2-ethylimidazolate zinc polymer. This comparison aims to discern the relative advantages and disadvantages of incorporating magnetic iron oxide nanoparticles in the contaminant removal process facilitated by a coordination polymer. Our findings reveal that even a minimal incorporation of iron oxide nanoparticles substantially enhanced the composite’s overall performance in pollutant adsorption.

## 1. Introduction

According to the World Health Organization (WHO), the most important chemical hazards in drinking water derive from arsenic, fluoride, and nitrate [1]. Nevertheless, contaminants of emerging concern such as pharmaceuticals, pesticides, fluoroalkyl substances, and microplastics are receiving more public attention [1]. After the recent sanitary emergency, there has been a notable increase in the accumulation of pharmaceutical contaminants in wastewater, as highlighted in recent studies [2]. Contaminants of emerging concern (CECs) are substances detected in the environment at low concentrations, yet there is not enough information regarding their potential harmful effects [3]. Diclofenac, a widely-used anti-inflammatory medication, has become a prominent example of these new contaminants, frequently detected in wastewater, surface water, and marine environments. Recent evidence has established the toxicity of diclofenac, as demonstrated in the well-documented vulture population decline case [4,5].

Metal oxides like TiO_2_ and ZnO have been extensively used as photocatalysts for the degradation of contaminants due to their semiconductor properties. Nevertheless, their activity is affected by the low affinity of the metal oxide surface towards organic molecules [3,6]. Metal Azolate Frameworks (MAFs) and highly porous materials and Zeolitic Imidazolate Frameworks (ZIFs) are considered new materials for contaminant removal from water by either sorption or photocatalytic degradation [7,8,9,10,11]. However, these porous materials have some drawbacks, such as pore collapse during solvent removal [12], longer equilibration times, and pore width-adsorbate size relationship must be adequate to allow adsorption on pore walls. More compact and robust coordination polymers with small particle sizes should not be discarded as options for water treatment; although smaller surfaces imply lower adsorption capacity, the affinity for organic pollutants should be better than that of metal oxides. The literature reports a variety of MOF composites, particularly those incorporating the widely studied ZIF-8 (a coordination polymer formed by 2-methylimidazole and zinc), mixed with materials such as metal oxides, fibers, metal nanoparticles, graphene, and enzymes [11,13,14,15].

ZIF-8 and its cobalt analogue, ZIF-67, are noted for their sodalite (SOD)-type topology and high specific area [16,17]. Despite the over 100 zeolite-like topologies identified for ZIFs, research predominantly focuses on the abovementioned structures for sorption or catalytic applications [9]. However, the small particle size poses challenges in separating them from water post-contaminant treatment, often necessitating centrifugation [3]. Magnetic ZIF-8 composites, which facilitate easier separation, are produced through multi-step synthesis processes where the coordination polymer is synthetized in the presence of magnetic particles [13,15]. Recent reports also indicate that the facile synthesis of magnetic Covalent Organic Frameworks (COFs) composites follows this strategy. Notably, while the solution-refluxing synthesis is scalable, obtaining completely homogeneous material is the most challenging step [18]. Simplifying and accelerating the synthesis of coordination polymers (MAFs or ZIFs) and their composites would greatly benefit their widespread application.

Other strategies for removing diclofenac and other nonsteroidal anti-inflammatory drugs from water include aqueous biphasic systems with ionic liquids (ILs), which have the disadvantages of using ILs solvents that are highly toxic and hard to re-use. Aiming to develop a more sustainable and cost-effective strategy, Almeida et al. reported the use of an aqueous biphasic system (ABS) composed of ionic liquids (ILs) and an aluminum-based salt, Al_2_(SO_4_)_3_ (flocculating agent), that reached up to 91% recovery of diclofenac, and experienced no detected losses on extraction efficiency and recovery [19].

In this work, we prepared a composite material combining MAF-32 and magnetic iron oxides (a mixture of maghemite and magnetite expressed as Fe_x_O_y_) using a straightforward one-pot methodology. This composite was developed for testing as a sorbent for the removal of diclofenac from water. The composite exhibits superparamagnetic behavior, a property that does not compromise the main matrix characteristics. Importantly, only a minimal amount of iron oxide (approximately 5%) is required to enable magnetic-assisted separation. We compared this composite with MAF-32 alone to demonstrate the benefits and drawbacks of incorporating magnetic particles into the material.

## 2. Materials and Methods

### 2.1. Chemicals

Ferrous ammonium sulfate hexahydrate (98%) was purchased from Meyer (Monterrey, Mexico) reagents, 2-ethylimidazole (98%), zinc sulfate heptahydrate (99%), zinc acetate dihydrate (99%), and diclofenac sodium salt (98%) were purchased from Sigma Aldrich (Darmstadt, Germany). All the reagents were used without further purification. Solutions were prepared with deionized water with a resistivity of 18 MΩ cm^−1^.

### 2.2. Preparation of MAF-32

Precursors 2-ethylimidazole (1.202 g, 12.5 mmol) and zinc acetate dihydrate (0.110 mg, 0.5 mmol) were dissolved in separated beakers in 25 and 10 mL of deionized water, respectively. Then, the ligand solution was added to the zinc salt solution without stirring. After a few seconds, a white precipitated started to form. The formation of the solid is slow, which can be followed by the cloudiness of the reaction mixture that disappears, after aging for one week. The white precipitated was filtered under vacuum and washed exhaustively with deionized water to remove the remanent acetate and ligand excess. The product was dried at 60 °C in a convection oven for 2 days to give a white powder 0.099 g, 77% of yield according to the zinc equivalents.

### 2.3. One-Pot Preparation of Composite Fe_x_O_y_/MAF-32

The ligand 2-ethylimidazole (1.202 g 12.5 mmol) was dissolved in 100 mL of deionized water in a round flask and N_2_ was bubbled to the solution for 30 min. Separately, 25 mL of deionized water solution with ferrous ammonium sulfate hexahydrate (0.050 g 0.127 mmol) and zinc sulfate heptahydrate (0.1438 g 0.5 mmol) was prepared and bubbled also with N_2_ for 15 min. Then the metal solution was added to the ligand solution under stirring and nitrogen atmosphere using a syringe. Immediately, a light green dispersion was formed. Nitrogen bubbling was maintained for 30 min. Stirring continued for two hours, after that the solution turned grey. Dispersion was allowed to settle for one night at room temperature and no coloration changes were observed. After decantation, the solid was filtered under vacuum and washed with deionized water. Finally, the solid was dried at 60 °C in a convection oven for one day yielding a light brown solid. The isolated product yielded 0.130 g.

### 2.4. Characterization

The materials were characterized by several analytical techniques: IR, X-ray powder diffraction, Mössbauer spectroscopy, XPS, magnetization vs. field curves, SEM, EDS, diffuse reflectance, and nitrogen adsorption.

IR measurements were taken in a Thermo-scientific FTIR Spectrophotometer Model Nicolet 6700 provided with a Smart iTR ATR accessory. The powder diffraction patterns were obtained in a Malvern Panalytical model Empyrean diffractometer with Cu Kα beam source. Rietveld refinement was performed with BGMN/Autoquan software version 4.2.22 [20] (user interface Profex 5.2.2 [21]) to calculate the percent composition of the composite using as reference MAF-32 [22], magnetite [23], and maghemite [24] files obtained from monocrystal X-ray diffraction data. Refinement was carried out in two separate phases: (i) MAF-32 and (ii) iron oxide. The BET specific area was measured at 77 K with a Micromeritics ASAP 2020 instrument; samples were degassed at 150 °C.

The ^57^Fe Mössbauer data were recorded at room temperature using a ^57^Co(Rh) source and a conventional constant acceleration spectrometer. The velocity scale was calibrated using a 6 μm-thick iron foil. The chemical isomer shifts were referred to the centroid of the room temperature Mössbauer spectrum of α-iron. The spectrum was computer-fitted. Magnetization curves were taken at a physical property measurement system DynaCool-9 in the range of 1.8 to 350 K and in the field range from −40 to 40 kOe.

X-ray photoelectron spectroscopy measurements were performed on three separate MAF batches to ensure data reproducibility. Sample preparation was performed under ambient atmosphere. Powders were dispersed in ethylene glycol to obtain a concentrated dispersion. The dispersion was drop-casted onto cleaned Si wafers and subsequently dried in a conventional oven at 50 °C for 30 min. Afterwards, the Si wafers were electrically grounded to the XPS sample bar by carbon tape. The XPS measurements were recorded with a Kratos Axis Ultra DLD system equipped with monochromatic Al Kα (hν = 1486.6 eV) X-ray source. During the measurements, pressure in the main chamber was kept below 1 × 10^−7^ bar. Charge compensation was carried out via a neutralizer running at a current of 7 μA, a charge balance of 5 V, and a filament bias of 1.3 V. The X-ray gun was set to 10 mA emission. Binding energies were referenced to C 1s peak arising from adventitious carbon with an emission energy of 284.8 eV. The C 1s, O 1s, N 1s, Fe 2p and Zn 2p core levels were recorded with an emission current of 10 mA, an accelerating voltage of 15 kV, and a pass energy of 80 eV. We collected three scans for iron, zinc, oxygen and nitrogen, and two scans for carbon. XPS analysis was performed with CasaXPS (version 2.3.22PR1.0). The U Touggard function was used for background subtraction. The XPS signals were fitted with the CasaXPS component fitting tool.

SEM micrographs were obtained using a Zeiss Auriga (Oberkochen, Germany) scanning electron microscope with a beam energy of 25 kV. The composite samples were drop-casted onto silicon wafers from hexane dispersions. EDS elemental information was also obtained using a Zeiss Auriga scanning electron microscope coupled to an EDS analyzer. Measurements were carried out using 25 kV electron beam energy. Semi-quantitative data analyses were performed using the EDAX APEX^TM^ 3.0 software.

### 2.5. Adsorption Measurements

The kinetic experiments were performed at 25 °C, placing 6.6 mg of MAF-32 and the composite in an amber vial, with 20 mL of a 20 mg L^−1^ diclofenac solution. Aliquots were taken from different vials at different times. In the diclofenac/MAF-32 system, vials were first shaken on an orbital shaker at 200 rpm for 5 min and then left static until aliquots were taken. For the diclofenac/composite system, shaking was performed continuously. The adsorption capacity was determined by the known equation: *q* = ((*C_i_* − *C_f_*)V)/*m*, where *q* is the adsorbed quantity, *C_i_* is the initial and *C_f_* the final concentration, V is the volume of diclofenac solution, and *m* is the adsorbent mass. The concentration changes in the solutions during the adsorption experiments were measured by RP-HPLC using an Agilent 1200 Infinity series chromatograph equipped with a Multiple Wave Detector 1260 Infinity II. The chromatographic column Zorbax C18 (4.6 × 100 mm, 3.5 μm) was used at 30 °C under isocratic conditions at a flow rate of 1 mL min^−1^, the eluent was a mixture of CH_3_CN and H_2_O (55:45) with 0.2% of formic acid.

The adsorption experiments were carried out at 25 °C in the concentration range within 1 mg L^−1^ and 40 mg L^−1^. The amount of adsorbent was 6.6 mg set in 20 mL of diclofenac solution in an amber vial. The systems were shaken for 5 min in the case of MAF-32 and for two hours in the experiments with the composite Fe_x_O_y_/MAF-32. The diclofenac concentration changes were monitored by RP-HPLC under the conditions described above. All experimental adsorption data were fitted by non-linear regression.

## 3. Results and Discussion

### 3.1. Compositional and Topological Identification

#### 3.1.1. Vibrational Spectroscopy

**Figure 1:**
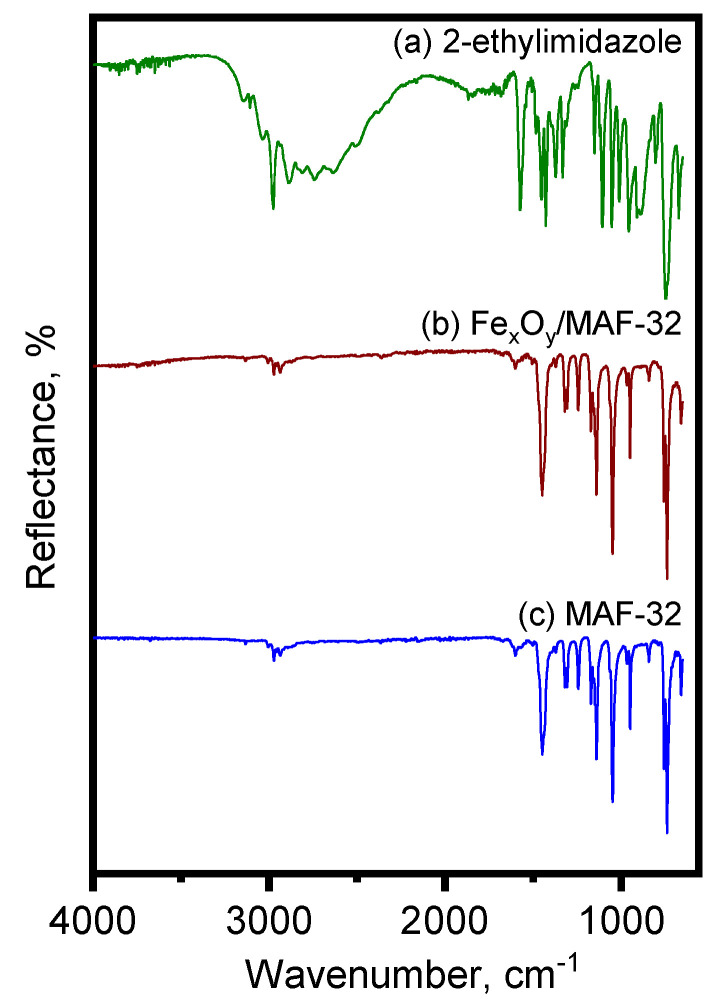
ATR-FTIR spectra of (a) 2-ethylimidazole, (b) Fe_x_O_y_/MAF-32, and (c) MAF-32.

Figure 1 displays the comparison of the ATR-FTIR spectra of MAF-32 and its iron composite. It shows the typical bands corresponding to the imidazole: C-H stretching at 3008, 2974, 2938 cm^−1^, C=C stretching at 1601 cm^−1^, C=N stretching at 1449, 1321, 1308 cm^−1^, C-H out-of-plane bending at 1049, 757, 739 cm^−1^, and for the ring deformation, an out-of-plane bending at 660 cm^−1^ [25]. The spectra of MAF-32 and Fe_x_O_y_/MAF-32 are almost identical; the main difference between the synthesis products and the free ligand is the absence of the broad band of N-H stretching in the high-energy region of the spectra and the N-H deformation mode at 1570 cm^−1^_,_ which corroborates the formation of the imidazolate anion. As shown in Figure 1, the iron oxide bands have such low intensity that they do not appear in the spectrum, which is congruent with the low content of the iron oxide in the composite. It is well-known that these kind of materials (coordination polymers based on imidazole with non-polar substituents) are highly hydrophobic. We corroborated this fact by noting the absence of the typical O-H stretching bands, which arise from water presence, thereby indicating the absence of water on both materials [26,27].

#### 3.1.2. Topology and Crystallinity Analyzed by Powder X-ray Diffraction

**Figure 2:**
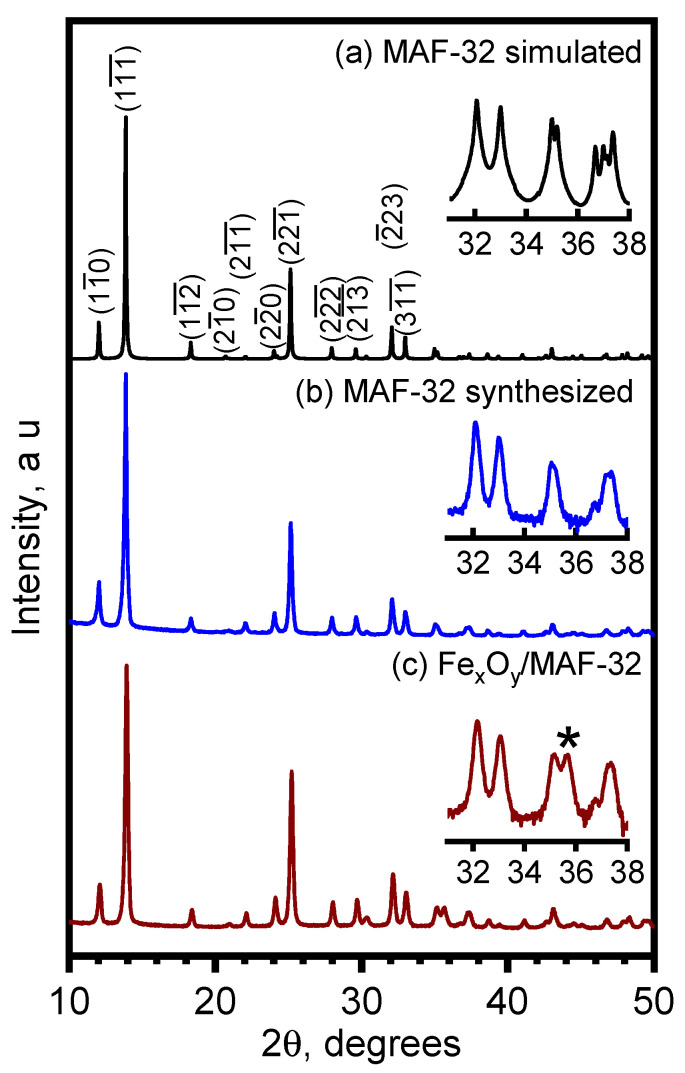
Diffraction patterns from 10 to 50 degrees of (a) simulated MAF-32, (b) synthesized MAF-32, and (c) Fe_x_O_y_/MAF-32. Inset: intensity amplification in logarithmic scale in the range from 31 to 38°.

The diffractogram of MAF-32 was simulated using Mercury 2024.1.0 software, utilizing the CIF file of the single-crystal structure retrieved from the CCDC database of a previously reported work [22]. The diffraction patterns of MAF-32 and Fe_x_O_y_/MAF-32 show the presence of characteristic peaks at 2θ from 10 to 35 degrees corresponding to the planes (1$\bar{1}$0), (1$\bar{1}\bar{1}$), (1$\bar{1}\bar{2}$), (2$\bar{1}$0), (2$\bar{1}\bar{1}$), (2$\bar{2}$0), (2$\bar{2}\bar{1}$), (2$\bar{2}\bar{2}$), (2$\bar{1}\bar{3}$), ($\bar{2}$23), and (3$\bar{1}\bar{1}$) of MAF-32. The comparison of the diffraction patterns obtained with the simulated MAF-32 agrees with the presence of the *qtz* topology. The crystallinity of pure MAF-32 is relatively higher than that of the composite, but they show almost identical diffractograms (Figure 2b,c). The principal difference is a very low intensity peak, marked with an asterisk in the inset of Figure 2c, at a diffraction angle of 2θ = 35.7° that corresponds, as expected, to the magnetite or maghemite (311) plane in the composite. In Figure 2b, the MAF-32 phase purity can be observed; the long reaction time yields only one of the three possible topologies that have been reported for zinc and 2-ethylimidazole coordination polymers [27]. Faster procedures to obtain MAFs has been reported, but even with the use of more complex methodologies, phase purity is not always achieved.

**Figure 3:**
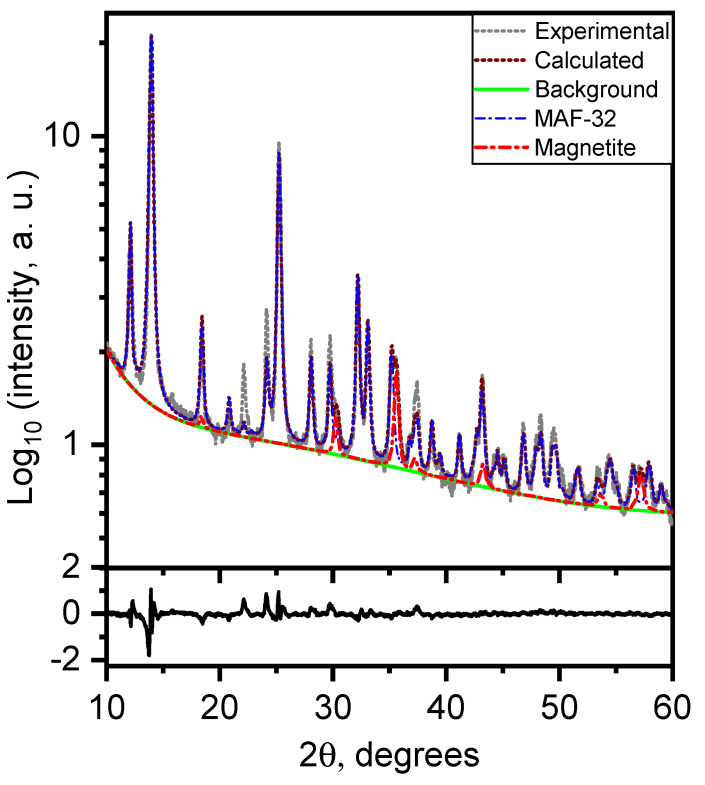
Rietveld refinement of the composite Fe_x_O_y_/MAF-32 (up). Difference between experimental and calculated patterns (below).

Regarding the iron oxide present in the composite, quantification of the magnetite/maghemite relationship was not conducted because other techniques surpass the XRD method for this purpose [28]. Therefore, we considered both phases of iron oxide in our approach to study the structure of the materials (Table 1). The Fe_x_O_y_/MAF-32 diffraction pattern fitting was approximately 95% MAF-32 and 5% of magnetite and maghemite, respectively (Figure 3). As shown in Table 1, the cell parameters were not significantly affected by the selection of one of these iron phases for the refinement and the low content agrees with the ATR-FTIR spectrum information. In addition, the crystallite mean size below 50 nm agrees with the existence of small domains that exhibit superparamagnetic behavior. The refinement of MAF-32 gives a bigger crystallite size (around 93 nm) and a wider distribution (k1 parameter in Table 1) than the MAF-32 phase in the composite Fe_x_O_y_/MAF-32.

### 3.2. Iron Oxide Phase Characterization and Magnetic Properties of the Composite

#### 3.2.1. Magnetite/Maghemite Identification by Mössbauer Spectroscopy

**Figure 4:**
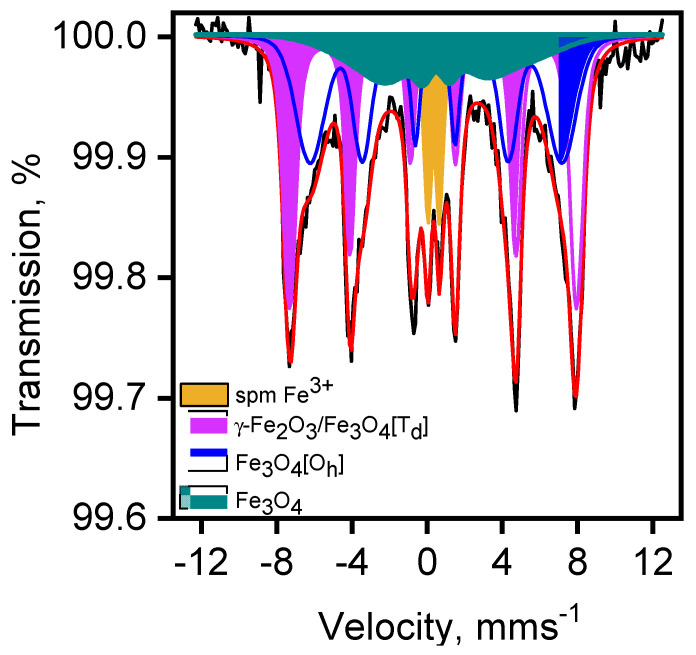
Mössbauer spectrum recorded at room temperature of Fe_x_O_y_/MAF-32. Red line shows the fitted spectrum.

The results of the ATR-FTIR spectroscopy and PXRD indicate that the main component of the composite Fe_x_O_y_/MAF-32 is the coordination polymer. To clarify the identity of the iron oxide phase, Mössbauer and magnetization experiments were performed. The room temperature Mössbauer spectrum recorded from Fe_x_O_y_/MAF-32 is depicted in Figure 4. The spectrum shows a main broad, asymmetric magnetic component and a smaller quadrupole contribution. The spectrum is characteristic of a system experiencing superparamagnetic relaxation associated to a distribution of iron oxide with small particle sizes. The spectrum was fitted to a model considering three different magnetic contributions and a quadrupole doublet. The hyperfine parameters obtained from the fit of the spectrum are collected in Table 2.

The narrower sextet (spectrum in magenta) has parameters which can be associated with maghemite (γ-Fe_2_O_3_) [30]. While the isomer and quadrupole shift of this sextet match those characteristics of this iron oxide, the hyperfine magnetic field is significantly smaller: 47.3 T vs. the “canonical” 49.9 T value [30]. It has been reported that the phenomenon of superparamagnetism is often reflected in a smaller value of the hyperfine magnetic field due to the occurrence of collective magnetic excitations [31,32]. This implies that, at room temperature, the size of the maghemite particles, although large enough as to show a well-developed magnetic sextet, is not sufficient to completely overcome the superparamagnetic relaxation, hence the smaller hyperfine magnetic field.

The second most intense magnetic component (blue) has an isomer shift (0.45 mms^−1^) which is quite large to be solely attributed to the presence of an Fe^3+^ oxide [30,33]. It is well-known that the room temperature Mössbauer spectrum of magnetite, Fe_3_O_4_, is composed of two different magnetic sextets, one accounting for the Fe^3+^ sitting in the tetrahedral sites of the spinel-related structure and a second one arising from the Fe^2+^/Fe^3+^ located in the corresponding octahedral sites in that structure [30,31,32,33]. Because of electron hopping, the chemical isomer shift of this second sextet (0.72 mms^−1^) is intermediate between that expected for an octahedral high spin Fe^3+^ ion (~0.35 mms^−1^) and that characteristic of an octahedral high spin Fe^2+^ ion (~1.1 mms^−1^); it is often referred to as the Fe^2.5+^ octahedral component of the Mössbauer spectrum of magnetite. Therefore, these results suggest that the broad blue sextet might arise from a fraction of small particle magnetite, as the increase in the value of the isomer shift compared to that expected for Fe^3+^ would suggest the participation of Fe^2+^ ions through an electron hopping mechanism. It has also been reported that superparamagnetic particles can not only show smaller hyperfine magnetic field values but also smaller isomer shifts [32,34]. Both circumstances are concurrent here, hence the assignment of this sextet as being due to superparamagnetic magnetite. However, we must not discard that, because the spectrum clearly indicates the occurrence of a distribution of particle sizes, a fraction of small particle maghemite (particles with sizes smaller than that responsible for the narrower magenta sextet) is also contributing to the blue sextet, providing a second, concomitant reason for its intermediate isomer shift value. The presence of magnetite was further confirmed by the presence of the third magnetic component (Figure 4 and Table 2, dark green component) which exhibits an isomer shift closer to that of the Fe^2.5+^ component of magnetite.

It is also a well-proven fact that in the absence of an external applied magnetic field, it is difficult to discern the sextet corresponding to the tetrahedral Fe^3+^ in magnetite from the Fe^3+^ sextet corresponding to maghemite. Since the Fe^2.5+^/Fe^3+^ sextet area ratio in the spectrum of stoichiometric magnetite is 1.9 [33], the fact that the (blue + green)/magenta sextet area ratio in the spectrum of the present sample is around 1.35, indicates the occurrence of a mixture of superparamagnetic magnetite and maghemite.

Finally, the quadrupole doublet (light orange in Figure 4) has hyperfine parameters characteristic of a high spin Fe^3+^ ion in octahedral oxygen coordination. Apart from corresponding to an octahedral high spin Fe^3+^ species, the nature of a doublet like this is quite unspecific, as it might arise from the presence of many different phases. For instance, it could correspond to a fraction of very small maghemite particles, where, due to superparamagnetic effects, the magnetic interactions have totally collapsed at room temperature, or to any other kind of microcrystalline/amorphous Fe^3+^ phase that went undetected by XRD.

The composite corresponds to a mixture of maghemite and magnetite, with a distribution of particle sizes ranging from small enough to show a completely paramagnetic spectrum at room temperature to large enough to produce a well-developed sextet with parameters close, but not quite identical, to those shown by bulk specimens. The data would be consistent with a magnetite core surrounded by a maghemite shell. In addition to the superparamagnetic effects due to the small sizes of the particles, the concomitant surface and interface effects would explain most of the phenomenology observed in the spectrum.

#### 3.2.2. X-ray Photoelectron Spectroscopy Studies

**Figure 5:**
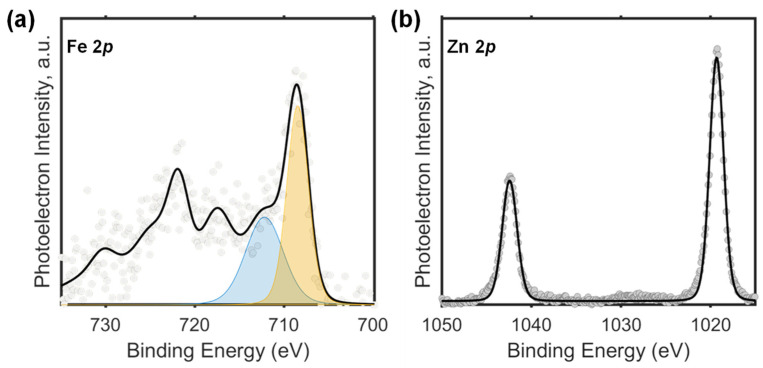
(**a**) Representative HR-XPS Fe *2p* region spectra of the composite material. The orange and blue shading depicts Gaussian peak fits to these data. The higher energy component (blue shading) is assigned to Fe^3+^ ions and the lower-energy component (orange shading) is assigned to Fe^2+^ ions. (**b**) Representative HR-XPS Zn *2p* region spectra of the composite material.

The surface chemistry of our composite was studied by X-ray photoelectron spectroscopy (XPS). XPS data were dominated mainly by the carbon and zinc signals arising from the MAF framework which encapsulates and surrounds the iron oxide nanoparticles. Given the characteristics of the composite and the low amount of iron oxide load (approximately 5%), it was difficult to obtain a Fe 2p spectrum with reasonable statistics as to extract unambiguous conclusions. Figure 5 shows representative Fe 2p and Zn 2p spectra. As explained above, the Fe 2p spectrum presents a poor signal-to-noise ratio. The main photoemission Fe 2p_3/2_ peak at 709 eV and the shoulder observed at 711.8 suggest the concomitant presence of Fe^2+^ and Fe^3+^, respectively. However, the quality of the data precludes its rigorous deconvolution making it difficult to separate both contributions (Figure 5a). The Zn 2p spectrum consists of a narrow spin-orbit doublet with binding energies (BEs) of Zn 2p_3/2_ = 1019 eV and Zn 2p_1/2_ = 1042 eV which is consistent with the presence of Zn^2+^ [8]. This analysis enhances our understanding of the surface characteristics and chemical states of iron and zinc in the composite, providing valuable insights into its chemical properties and potential applications.

#### 3.2.3. Magnetic Properties of the Composite Fe_x_O_y_/MAF-32

**Figure 6:**
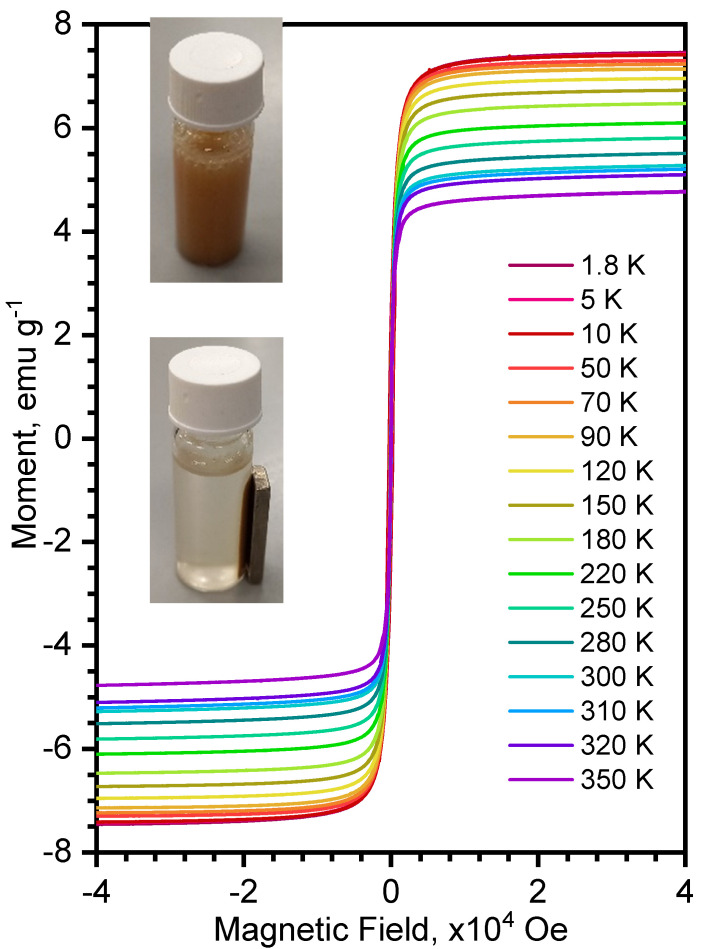
Magnetization (M) versus applied magnetic field at different temperatures (1.8–350 K) of Fe_x_O_y_/MAF-32. Inset: initial dispersion (upper picture) and the solid magnetically separated (lower picture).

The magnetic curves (M vs. H) of the composite Fe_x_O_y_/MAF-32 are shown in Figure 6. The curves do not show a hysteresis loop (Hc value close to zero) which is a confirmation of the superparamagnetic behavior of the composite. The magnetization saturation (Ms) changed from 4.79 to 7.45 emu g^−1^ in the studied temperature range. These low values are due to the small percent of iron oxide (~5%) in the composite. The magnetic response to a magnetic field was tested by placing a commercial magnet beside a vial with an aqueous dispersion of the composite. The inset in Figure 6 shows the initial dispersion, which becomes totally agglomerated after 90 s, while MAF-32 remains dispersed in an aqueous system for days until sedimentation of the solid is complete. It can be inferred that all the coordination polymer particles have magnetic iron oxide particles embedded in their structures. The low value of saturation magnetization agrees with a fraction of Fe_3_O_4_ nanoparticles (92 emu g^−1^). Notably, this low amount is enough for the enhanced assisted separation, which is the main purpose of the addition of iron oxide. 

### 3.3. Morphology and Elemental Composition of the Surface

**Figure 7:**
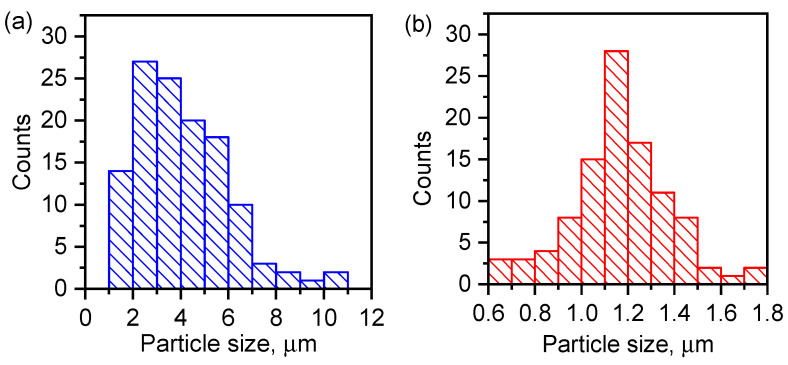
Particle size distribution of (**a**) MAF-32 and (**b**) composite Fe_x_O_y_/MAF-32.

**Figure 8:**
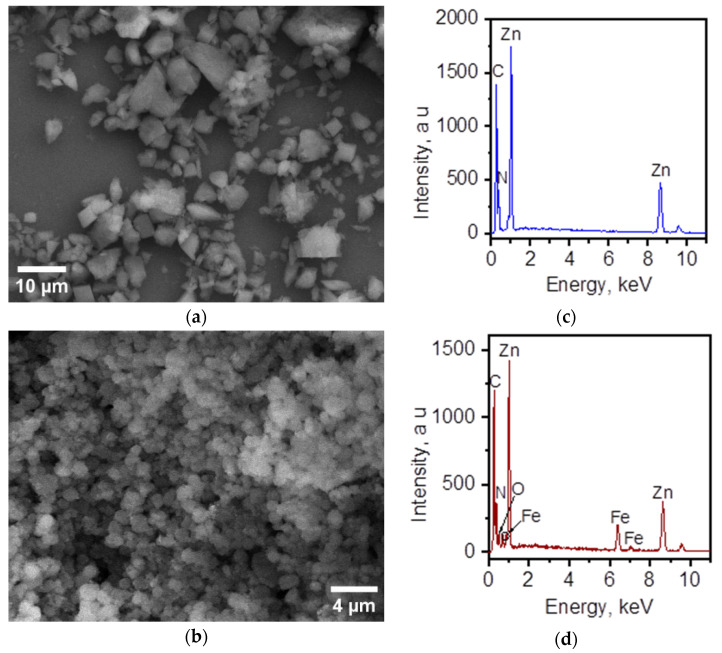
Representative SEM micrographs of (**a**) MAF-32 (scale bar = 10 μm) and (**b**) nanostructured composite (scale bar = 4 μm). EDS spectrum of (**c**) MAF-32 and (**d**) the composite material showcasing the presence of all expected elements in their structures.

The morphology and composition of the composite and the MAF were analyzed by scanning electron microscopy (SEM) and energy-dispersive X-ray spectra (EDS). Differences in the particle size of the MAF and the composite were observed by SEM. Figure 7 shows that the particle size is lower for the composite material and the size distribution is narrow and centered at 1.2 μm. MAF-32 presents a more dispersed particle size. Figure 8b shows the overall polyhedron/sphere-like morphology of the obtained composite. Figure 8d displays the EDS spectra of this composite material confirming the presence of all expected elements.

### 3.4. Optical Properties: MAF vs. MAF Composite

**Figure 9:**
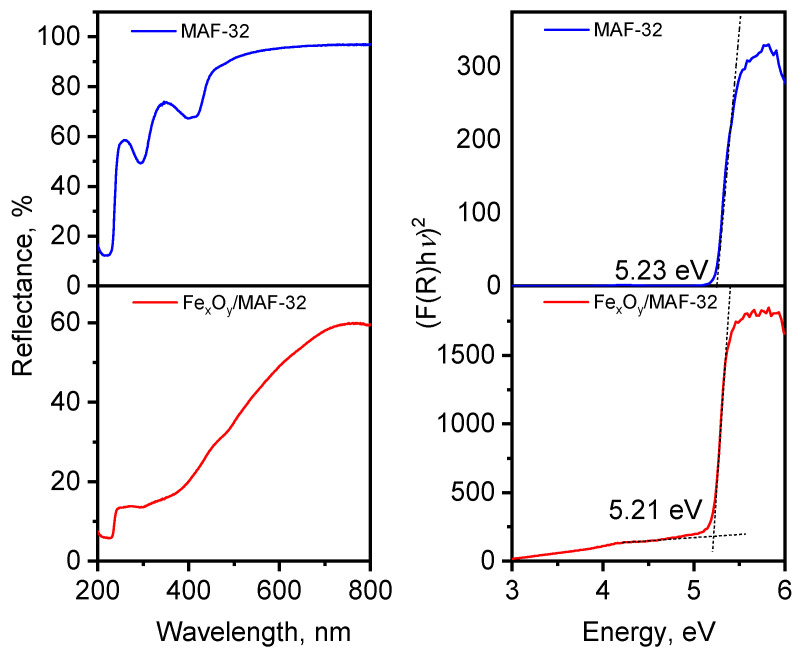
Solid-state optical diffuse-reflection spectra of MAF-32, and Fe_x_O_y_/MAF-32 (left side). Tauc’s plot of MAF-32, and Fe_x_O_y_/MAF-32 (right side).

The reflectance diffuse spectra shown in Figure 9 display the comparison of the *E_g_* value estimated by making the baseline correction to the Tauc’s plot method [35] and taking the first derivative to find the inflection point. The band gap energy value of the composite Fe_x_O_y_/MAF-32 of 5.21 eV is basically the same as that of MAF-32 (5.23 eV), which implies that the material is a composite, and it is not an iron-doped material. This distinction should be clarified because the synthesis was realized in one step from a homogeneous solution of zinc and iron salts. These *E_g_* values are very similar to those obtained by theoretical calculations [36,37]. Also, the *E_g_* values resemble the experimental one of ZIF-8, of approximately 5 eV [8], since the ligands 2-ethylimidazole and 2-methylimidazole used in the synthesis of MAF-32 and ZIF-8, respectively, are very similar ligands in terms of electron density donation. Additionally, the composite Fe_x_O_y_/MAF-32 shows a broad absorption band at energies below 5 eV corresponding to the absorption of iron oxides. Because of the width of that band, it is not possible to extrapolate the linear region to determine the *E_g_* value; however, the reported value for magnetite and maghemite is close to 2 eV [38,39]. A larger absorption at low energy in the composite is more evident in the absorbance spectra (not shown but can be inferred from the reflectance spectrum) due to the ability of iron oxide to absorb in the visible region of the spectra [3].

### 3.5. Textural Properties

**Figure 10:**
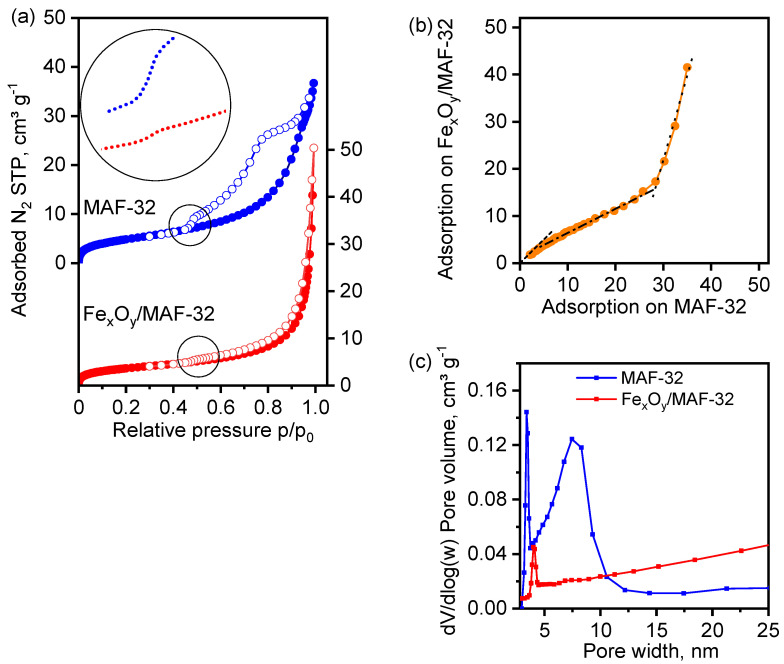
(**a**) Nitrogen adsorption-desorption isotherms of MAF-32 and Fe_x_O_y_/MAF-32. Full symbols: adsorption, empty symbols: desorption. Inset: desorption isotherms in the 0.4 < p/p_0_ < 0.6 range. (**b**) Comparative isotherm of nitrogen adsorbed (cm^3^ g^−1^) by the two materials. (**c**) Pore size distribution.

Nitrogen adsorption isotherms were measured to evaluate the specific surface area of the samples (Figure 10). The MAF-32 sample shows a type IV adsorption isotherm with a BET surface area of 17.9 m^2^g^−1^, where the hysteresis loop closing at p/p_0_ > 0.4 indicates the material presents pores in the mesopore range. Several slopes in the desorption isotherm within the hysteresis loop move the isotherm away from a characteristic behavior and might reflect a complex system of pores. The composite Fe_x_O_y_/MAF-32 shows a type IIb adsorption isotherm with non-rigid pores or cavities between agglomerates and a BET specific surface area of 13.3 m^2^g^−1^. Nitrogen was less adsorbed by the composite than by the MAF probably due to pore blocking, and both samples exhibit a sub-step at p/p_0_ 0.4 only in the desorption isotherm, and less intense in the composite. This observation suggests that it is not associated with changes in interglobular voids but with the polymer matrix. In addition, the absence of mesopores in the pore size distribution and the decrease in micropore volume (Figure 10c) suggest pore blocking caused by the small magnetic particles. The specific surface is slightly reduced during the process of integration of the magnetic particles in the coordination polymer matrix. However, the decrease is not substantial (compared with the acquired magnetic properties). The low magnitudes of the specific surface area are congruent with the formation of a compact phase. The changes in the nitrogen adsorption isotherm of the iron-MAF composite with respect to the MAF indicate that the textural characteristics are sensitive to the presence of the magnetic particles. In the comparative graph (Figure 10b), three linear segments are shown in the adsorption curve. The first shows that the adsorption isotherms of both materials agree only at very low pressures. Then, the lower slope in the mesopore region indicates less adsorption ability of the composite. The opposite behavior is observed in the high-pressure region. The pore size distribution of the MAF-32 in Figure 10c indicates a bi-disperse system of pore sizes, with *quasi*-micropores centered at 3.4 nm and small mesopores with a wide distribution centered at 7.5 nm. The micropores of the composite are slightly larger, 4 nm, and their volume contributes less to the total adsorbed volume. The crystalline structure of the coordination polymer with the compact *qtz* topology has a maximum pore diameter of 0.17 nm, which is smaller than the diameter of the nitrogen gaseous molecule (0.3 nm). Therefore, adsorption occurred on the external surface, inside pores formed by defects in the materials and interglobular cavities in the two cases. 

### 3.6. Adsorption of Diclofenac by MAF-32 and Fe_x_O_y_/MAF-32

**Figure 11:**
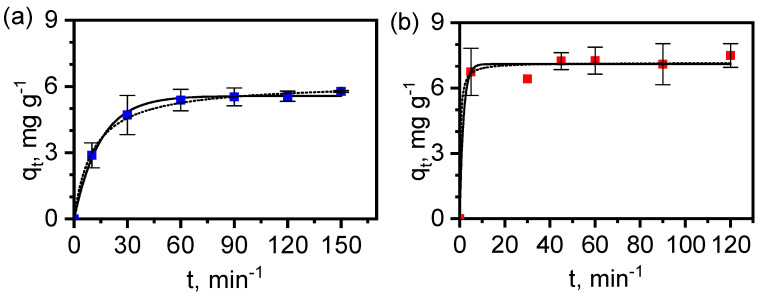
Kinetic curves of diclofenac adsorption experiments at 25 °C on (**a**) MAF-32 and (**b**) Fe_x_O_y_/MAF-32. Symbols: experimental values. Continuous line: *pseudo*-first order fitting and dotted line: *pseudo*-second-order fitting.

Kinetic results of diclofenac adsorption at 25 °C from aqueous solution were fitted to both *pseudo*-first and *pseudo*-second-order rate law equations (Figure 11), the equations used to fit experimental data are shown below, respectively:

(1) $$q_{t}=q_{e}(1-e^{-k_{1}t})$$

(2) $$q_{t}=\frac{\;q_{e}^{2}k_{2}t}{1+{q_{e}k}_{2}t}$$

The results are summarized in Table 3. The MAF-32 data fit equally well with both the *pseudo*-first-order law as with the *pseudo*-second-order law. However, the calculated adsorbed amount at equilibrium *q_e,calc_* is closer to the experimental data for the *pseudo*-first order than for the *pseudo*-second order. A different situation concerns the data fit for Fe_x_O_y_/MAF-32, since the correlation coefficient is acceptable for both orders that yield calculated *q_e,calc_* similar to the experimental *q_e,exper_*. Hence, in this case, more studies are needed for the exact determination of the kinetic order during adsorption under the given conditions. It should be noted that the kinetic constants *k*_1_ and *k*_2_ are larger for the composite, which implies that the adsorption equilibrium is reached faster and the affinity towards diclofenac improves with the presence of magnetic metal oxide particles. 

**Figure 12:**
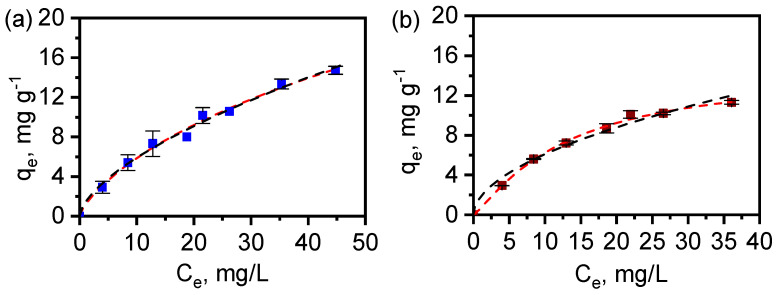
Diclofenac adsorption isotherms on (**a**) MAF-32 and (**b**) Fe_x_O_y_/MAF-32. Red dash lines correspond to the Langmuir-Freundlich fitting and black dash lines correspond to the Freundlich fitting.

The diclofenac adsorption isotherms were measured also in aqueous solution (Figure 12). They were adjusted to the Langmuir-Freundlich (Equation (3)) and Freundlich (Equation (4)) equations (shown below) and the resulting parameters are shown in Table 4.

(3) $$q_{e}=\frac{q_{m}k_{LF}C_{e}^{n}}{1+k_{LF}C_{e}^{n}}$$

(4) $$q_{e}=k_{F}C_{e}^{n}$$

The calculated curves fitted well with experimental values, and the maximal adsorption capacity (*q_m_*) predicted for MAF-32 with the Langmuir-Freundlich equation exceeded the experimental measurement range, as the adsorbent did not achieve saturation. In contrast, the *q_m_* value of 14.7 mg g^−1^ was obtained for the composite Fe_x_O_y_/MAF-32 and it is similar to the experimental value. This equation reflects a clear different heterogeneity of both materials since *n* < 1 for MAF-32 and *n* > 1 for Fe_x_O_y_/MAF-32. However, it is not possible to make a direct comparison of both materials by observing the parameters from the Langmuir-Freundlich equation. Then, when applying the Freundlich equation, the fitting parameters of the adsorption isotherm for both MAF-32 and Fe_x_O_y_/MAF-32 exhibited similar fits. The comparison of the constant *k_F_* suggests a higher affinity towards diclofenac for the composite Fe_x_O_y_/MAF-32 than for MAF-32, despite its lower adsorption capacity. The last implies that certain cavities in the coordination polymer are obstructed by iron oxide particles, changing not only the specific surface area but also the affinity towards diclofenac in the composite. The different adsorption capacity: 12.9 mg g^−1^ of MAF-32 and 11.2 mg g^−1^ of the composite taken at *C_e_* 35 mg L^−1^ is explained by the general decrease of pore volume and mesopores in the composite, as it was shown in the pore size distribution (Figure 10c).

**Figure 13:**
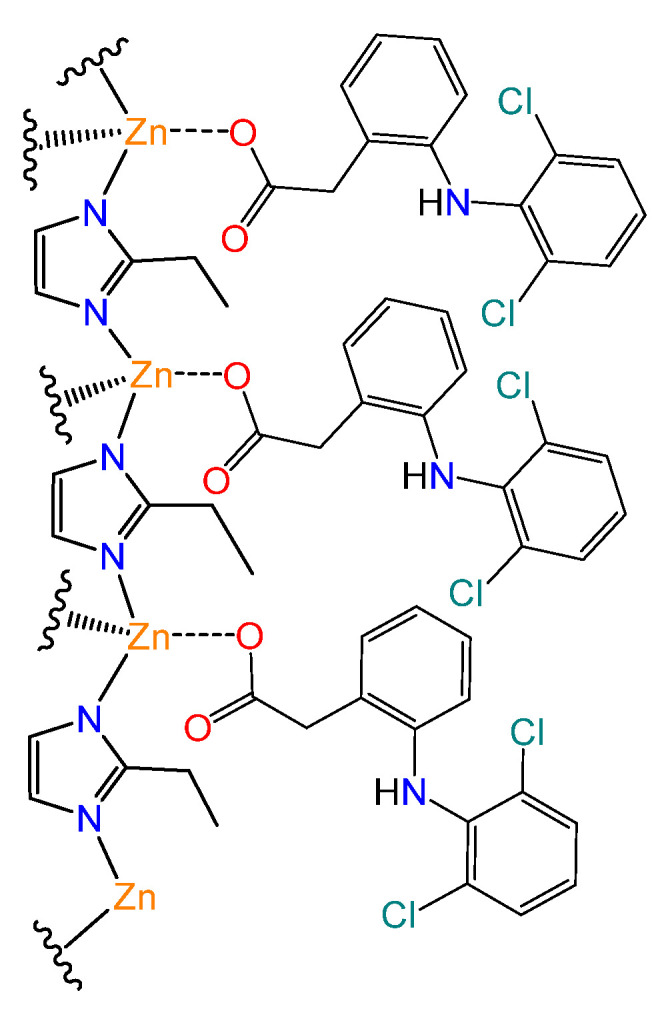
Interaction mechanism proposal for the adsorption of diclofenac on the coordination polymer surface.

According to the literature, the surface charge of ZIF-8 should be similar to that of MAF-32, because the basic pK of 2-ethylimidazole is 8.0, which is very close to that of 2-metylimidazole [40]. The *E_g_* values also indicate that MAF-32 is similar in terms of electron density at the zinc atoms and the imidazolate ligands. The solutions of concentration 20 ppm for the kinetic studies and 60 ppm for the isotherms of sodium diclofenac were slightly acidic (pH 5); therefore, the surface of the material should be positively charged (point of zero charge of ZIF-8 is 9.3 [41]). At the same pH of 5, the microspecies of diclofenac with 91% abundance is the one with negative charge (the carboxylate) [42]. Consequently, the interaction of a positively charged surface of the material and the negatively charged diclofenac carboxylate was proposed as the principal interaction during adsorption (Figure 13). The formation of an inner sphere complex has been proposed as the adsorption mechanism in similar systems [41]. The pore width of the crystalline structure with *qtz* topology has a maximum pore diameter smaller than the minimum projection diameter of diclofenac molecule (0.92 nm) [42]. Therefore, the adsorption was accomplished on the external surface, pores formed by material defects, and interglobular cavities of the MAF-32 and the composite, similar to the case of nitrogen adsorption. When the surface of Fe_x_O_y_/MAF-32 is measured using the monolayer capacity *q_m_* and the minimum (4.1 nm^2^) and maximum (7.9 nm^2^) projection area of diclofenac [42], the values are 8.7 m^2^ g^−1^ and 16.7 m^2^ g^−1^, respectively. The latter corroborates the approach shown in Figure 13, as the *S*_BET_ gives an intermediate value of 13.3 m^2^ g^−1^.

The adsorption of diclofenac on Fe_x_O_y_/MAF-32 is low compared to the capacity of other materials. Recently, a magnetic-GO/ZIF-8/g-AlOOH-NC composite (three components) with the highest monolayer adsorption capacity, *q_m_* = 2594 mg g^−1^ was reported [43]. However, these materials have very different compositions and natures, and their performance is unparalleled. GO and other carbon-based materials have a greater affinity to organic molecules, like various porous materials for the diclofenac adsorption summarized in this work. A similar material based on imidazolate, ZIF-67 functionalized with cetyltrimethylammonium bromide, with a specific surface area of 817 m^2^ g^−1^, adsorbed 38 mg g^−1^ of diclofenac, similar to Fe_x_O_y_/MAF-32. For ZIF-67, the pore width was the determining factor above the high specific surface because the diclofenac molecule could not enter its cavitites [10]. Large-size molecules can be adsorbed on either microporous or nonporous materials with similar performance if the adsorbate size exceeds the pore size. Adsorption to nonporous materials allows for understanding the adsorption mechanisms on materials with the same composition but different topologies.

## 4. Conclusions

The comprehensive characterization of our synthesized materials, utilizing techniques such as ATR-FTIR, N_2_ adsorption, powder XRD, Mössbauer spectroscopy, SEM, EDS, diffuse reflectance, XPS, and magnetization, reveals that magnetic particles (a mix of magnetite and maghemite) are effectively embedded within the MAF-32 matrix. This incorporation stabilizes the small iron oxide domains, necessary to provide the superparamagnetic behavior to our composite. Our findings indicate that while Fe_x_O_y_/MAF-32 composite exhibits a lower diclofenac adsorption capacity compared to MAF-32, likely due to iron oxide particles occupying some of the cavities, it exhibits a higher affinity for diclofenac, as evidenced by the analysis of the adsorption constants. A mechanism of inner sphere is the most reasonable due to the chemical nature of the surface and the adsorbate at a pH value lower than the point of zero charge. Moreover, the composite can be easily recovered with the assistance of a common commercial magnet. The thorough characterization and adsorption experiments were important to evaluate the effect of adsorption, even if modest, and could be involved in photocatalytic studies. The analysis of differences in the maximum adsorption capacity of nitrogen and diclofenac on Fe_x_O_y_/MAF-32 revealed that micro and mesopore blocking are the main reason for the decrease. It is expected that the difference will be negligible in composites with high specific surface materials and the same 5% of magnetic particles. In summary, the presence of superparamagnetic particles improves the adsorption performance of the MAF, and the “one-pot” synthesis method should be considered as a straightforward approach for the synthesis of MAF and MOF composites in aqueous solutions.

## Figures and Tables

**Table 1:** Selected refinement parameters obtained by the Rietveld method of MAF-32 and the composite, considering magnetite or maghemite for the refinement. k1 is a normalized parameter of crystallite size broadening (smaller k1 values correspond to a wide distribution [29]), *R_wp_* is the weighted residual square sum, *R_exp_* is the possible minimum value for *R_wp_*, and *GoF* is the goodness-of-fit.

Parameters	MAF-32	Composite Fe_x_O_y_/MAF-32		Composite Fe_x_O_y_/MAF-32	
		MAF-32	Magnetite Fe_3_O_4_	MAF-32	Maghemite Fe_2_O_3_
Space group	P6_4_	P6_4_	$F4_{1}/d\bar{3}$_2_/m	P6_4_	P4_1_32
Cell parameters					
*a* (nm)	0.8483 (2)	0.8497 (4)	0.8395 (4)	0.8498 (4)	0.8397 (4)
*c* (nm)	1.2852 (3)	1.2858 (6)	–	1.2859 (6)	–
k1	0	1	1	1	1
Crystallite size (nm)					
<100>	93 (3)	48.3 (9)	24 (1)	48.3 (9)	23 (1)
<010>	93 (3)	48.3 (9)	24 (1)	48.3 (9)	23 (1)
<001>	126 (11)	77 (8)	24 (1)	78 (8)	23 (1)
Composition (%)		95.2 (2)	4.8 (2)	94.3 (3)	5.7 (3)
R_wp_	9.03	7.56		7.50	
R_exp_	4.53	2.87		2.87	
GoF	1.99	2.63		2.61	

**Table 2:** Hyperfine parameters obtained from the fit of the spectrum recorded at room temperature from sample Fe_x_O_y_/MAF-32.

Site/Species	δ (mms^−1^)	Δ or 2ε (mms^−1^)	H (T)	Area (%)
spm Fe^3+^ (Doublet)	0.34	0.63	--	8
γ-Fe_2_O_3_/Fe_3_O_4_[T_d_] (Sextet 1)	0.32	−0.01	47.3	39
Fe_3_O_4_[O_h_] (Sextet 2)	0.45	0.04	41.5	36
Fe_3_O_4_ (Sextet 3)	0.66	0.39	26.2	17

δ: isomer shift; Δ: quadrupole splitting (doublet); 2ε: quadrupole shift (sextets); H: hyperfine magnetic field.

**Table 3:** Experimental and calculated kinetic parameters for the adsorption of diclofenac (20 mg L^−1^) using a *pseud*o-first-order and *pseudo*-second-order rate law fitting at 25 °C.

	MAF-32	Fe_x_O_y_/MAF-32
*Pseudo*-first-order		
*q_e,exper_* (mg g^−1^)	5.8	7.5
*q_e,calc_* (mg g^−1^)	5.6 ± 0.1	7.1 ± 0.2
*k*_1_ × 10^3^ (s^−1^)	1.1 ± 0.1	9.9 ± 4.0
*R* ^2^	0.997	0.985
*Pseudo*-second-order		
*q_e,calc_* (mg g^−1^)	6.2 ± 0.1	7.2 ± 0.2
*k*_2_ × 10^3^ (g mg^−1^ s^−1^)	0.2 ± 0.02	6.2 ± 5.5
*R* ^2^	0.998	0.986

**Table 4:** Langmuir-Freundlich and Freundlich parameters for diclofenac adsorption from aqueous solution at 25 °C on MAF-32 and the composite.

	MAF-32	Fe_x_O_y_/MAF-32
Langmuir-Freundlich		
*q_m_* (mg g^−1^)	55.5 ± 60.9	14.7 ± 1.4
*k_LF_* (mg g^−1^)	0.02 ± 0.02	0.05 ± 0.01
*n*	0.75 ± 0.17	1.19 ± 0.14
*R_LF_* ^2^	0.992	0.997
Freundlich		
*k_F_* (mg g^−1^) (L mg^−1^)^1/*n*^	1.4 ± 0.2	1.8 ± 0.3
*n*	0.62 ± 0.03	0.53 ± 0.05
*R_F_* ^2^	0.991	0.982

## Data Availability

Data are contained within the article.
